# ‘Public enemy no. 1’: Tobacco industry funding for the AIDS response

**DOI:** 10.1080/17290376.2016.1164617

**Published:** 2016-03-29

**Authors:** Julia Smith, Sheryl Thompson, Kelley Lee

**Affiliations:** ^a^ PhD Peace Studies, is a Postdoctoral Fellow in the Faculty of Health Sciences, Simon Fraser University, Burnaby, BC, Canada; ^b^ BA (candidate), is a Research Assistant in the Faculty of Health Sciences, Simon Fraser University, Burnaby, BC, Canada; ^c^ DPhil International Relations, is Professor in Global Health in the Faculty of Health Sciences, Simon Fraser University, Burnaby, BC, Canada; ^d^ is Professor in Global Health Policy in the Department of Global Health and Development, London School of Hygiene & Tropical Medicine, London, UK

**Keywords:** HIV, AIDS, donors, corporations, WHO, tobacco industry, SIDA, donateurs, l'indsutrie de tabac, l'Organisation mondaile de la santé

## Abstract

This article analyzes the history of tobacco industry funding for the AIDS response – a largely ignored aspect of private donor involvement. Primary documents from the Legacy Tobacco Documents Library and AIDS organizations are analyzed, alongside existing literature on the tobacco control and AIDS responses. Research on the tactics of transnational tobacco companies has documented how they have used various charitable causes to subvert tobacco control efforts and influence public health policy. This raises questions, which this paper seeks to answer, about if donations by tobacco companies to AIDS organizations have been used for similar means, and if so how AIDS organizations have responded to tobacco industry overtures. Two examples illustrate how tobacco companies initially tried to use the AIDS response to counter tobacco control measures: (1) During the 1990s, Philip Morris, one of the largest corporate donors of the AIDS response in the USA, used its connections with AIDS organizations to create competition for health resources, improve its reputation, and market tobacco products to the LGBT community; (2) In both Latin America and sub-Saharan Africa, Philip Morris and British American Tobacco championed the AIDS response in order to delegitimize efforts to develop the World Health Organization’s Framework Convention on Tobacco Control. However, from the late 1990s onwards, AIDS organizations began to refuse tobacco funding and partnerships – though these policies have been not comprehensive, as many tobacco companies still fund programs in sub-Saharan Africa. The article concludes that tobacco companies aimed to exploit competition between health issues, and use the high-profile AIDS response to improve their reputation and market access. However, AIDS organizations, adhering to broader health goals and drawing on extensive resources and networks, were able to shut the tobacco industry out of much of the response, though pockets of influence still exist. This demonstrates the importance of co-operation and policy convergence across health sectors and suggests that tobacco control advocates, and other charitable sectors that receive funding from the tobacco industry, may be able to draw lessons from the experiences of AIDS organizations.

## Résumé

Cet article analyse l’histoire du financement de la lutte contre le SIDA par l’industrie du tabac – un aspect de l’implication de donateurs privés largement ignoré. Sont analysés des documents primaires provenant de la *Legacy Tobacco Documents Library* (documents internes de l'industrie du tabac) et d’organismes de lutte contre le SIDA, ainsi que de la littérature existante sur la lutte anti-tabac et la réponse au SIDA. Des recherches antérieures sur les tactiques des multinationales du tabac ont documenté comment elles ont utilisé diverses causes caritatives pour subvertir les efforts anti-tabac et influencer les politiques de santé publique. Cela soulève des questions auxquelles cet article s’efforce de répondre: les donations des entreprises du tabac aux organismes de lutte contre le SIDA ont-elles été faites à des fins similaires? Dans l’affirmative, comment les organismes de lutte contre le SIDA ont-elles répondu aux ouvertures de l’industrie du tabac? Deux exemples montrent que des entreprises du tabac ont initialement essayé d’utiliser la réponse au SIDA pour contrer des mesures anti-tabac : 1) Dans les années 1990, Philip Morris, l’un des plus grands donateurs pour la lutte contre le SIDA du secteur privé, a utilisé ses contacts avec les organismes de lutte contre le SIDA pour créer de la concurrence sur les ressources pour la santé, améliorer sa réputation, et commercialiser des produits du tabac auprès de la communauté homosexuelle; 2) En Amérique latine et en Afrique subsaharienne, Philip Morris et British American Tobacco se sont engagés en faveur de la réponse au SIDA pour délégitimer les efforts visant à développer la convention-cadre de l'Organisation mondiale de la santé (OMS) pour la lutte anti-tabac. Toutefois, à partir de la fin des années 1990, des organismes de lutte contre le SIDA ont commencé à refuser des financements et partenariats liés au tabac – cependant ces politiques n’ont pas été appliquées partout: de nombreuses entreprises du tabac continuent à financer des programmes en Afrique subsaharienne. Cet article conclut que des entreprises du tabac ont eu pour objectif d’exploiter la concurrence entre les questions de santé, et d’utiliser la réponse au SIDA – qui bénéficie d’une très grande visibilité – pour améliorer sa réputation et son accès aux marchés. Néanmoins, les organismes de lutte contre le SIDA, en adhérant à des objectifs plus larges en matière de santé et en s’appuyant sur des ressources et réseaux considérables, sont parvenus à exclure l’industrie du tabac d’une grande partie de la réponse, bien que certaines influences persistent. Cela montre l’importance de la coopération et de la convergence des politiques à travers les secteurs, et suggère que les défenseurs de la lutte contre le tabac, et d’autres secteurs caritatifs qui reçoivent des financements de l’industrie du tabac, pourraient être en mesure de tirer des leçons des expériences d’organismes de lutte contre le SIDA.

## 1. Introduction

This article analyzes the history of tobacco industry funding for the AIDS response – a largely ignored aspect of private donor involvement. Research on private donor involvement in the HIV/AIDS response to date has largely focused on the role of pharmaceutical companies (Flanagan & Whiteman 2007; Lee & Kohler 2010; Leisinger 2005), as well as the creation of public–private partnerships to mobilize innovative financing (Buse & Harmer 2007; Ramiah & Reich 2006; Williams & Rushton 2011). Meanwhile, the tobacco control literature only refers to AIDS initiatives in passing. An article on Philip Morris’ (PM) attempts to generate legitimacy by working with health organizations refers to partnerships with AIDS Service Organizations (ASOs), but does not specifically discuss the dynamics between the tobacco industry and ASOs (McDaniel 2006). Drabble (2000) documents how PM targeted lesbian, gay, bisexual and transgender (LGBT) communities with donations to related causes, but does not analyze the AIDS response in depth. Other research (Mamudu, Hammond & Glantz 2008; Muggli & Hurt 2003; Rimmer 2005) briefly refers to tobacco industry sponsorship of specific AIDS events, but does not explore engagement in the AIDS response as an ongoing strategy employed by transnational tobacco companies (TTCs). The literature from other health sectors documents how TTCs have used charitable causes to subvert tobacco control efforts (Mackenzie & Collin 2008). For example, in 1999, British American Tobacco (BAT) sponsored the creation of the Beijing Liver Foundation for the specific purposes of diverting government attention away from tobacco-related illnesses (Muggli, Lee, Gan, Ebbert & Hurt 2008). This raises questions, which this paper seeks to answer, about if donations by tobacco companies to the AIDS response were used for similar means. Furthermore, both the tobacco control and AIDS literature focuses on the role of tobacco companies as donors, not those organizations that are recipients of funding, and therefore fails to consider how organizations and charitable sectors targeted by tobacco funding respond to the ethical dilemma of receiving resources from an industry whose products are harmful to public health.

In order to contribute to filling these gaps in current research on private donors to the AIDS response and AIDS organizations’ policies in regard to the tobacco industry, this article demonstrates how TTCs initially tried to use the AIDS response to counter tobacco control measures, and then how actors in the AIDS response developed policies to restrict the participation of TTCs in global health. The article finds that TTCs aimed to exploit competition between health issues and use the high-profile AIDS response to improve their reputation and market access. However, AIDS organizations, adhering to broader health goals while drawing on extensive resources and networks, were able to shut the tobacco industry out of much of the response, though pockets of influence still exist. This demonstrates the importance of co-operation and policy convergence across health sectors.

In addition to contributing to the literature on AIDS funding, this article adds to understanding of the challenges of and opportunities for implementing Article 5.3 of the Framework Convention on Tobacco Control (FCTC), ‘on the protection of public health policies with respect to tobacco control from commercial and other vested interests of the tobacco industry’ (WHO 2014). The FCTC, introduced in 2003 and now signed by 168 states, aims to reduce tobacco-related illness and death worldwide and limit the influence of the tobacco industry in public health policy. This article explores how AIDS organizations, as potential recipients of tobacco industry funding, make use of the norms embedded in the FCTC, contributing to its legitimacy. It suggests that tobacco control advocates, and other charitable sectors that receive funding from the tobacco industry, may be able to draw lessons from the experiences of AIDS organizations.

Following a brief description of the research methodology, the article provides two examples of tobacco industry involvement in the AIDS response: PM’s support for the early AIDS response in the United States (US), and PM’s and BAT’s efforts to delegitimize the World Health Organization’s (WHO) FCTC process by arguing that AIDS was a more important global health concern than tobacco control. It then documents the measures AIDS organizations took to restrict tobacco industry interference, and the limitations on these efforts, particularly in sub-Saharan Africa. It concludes with policy implications for the AIDS response specifically, and global health governance more generally.

## 2. Methods

The research began with a review of secondary literature to identify key processes, actors, events, and initiatives in the relationship between organizations involved in the AIDS response and tobacco industry. The Legacy Tobacco Documents Library, a collection of around 14 million industry documents that were released following US litigation, was then searched using keywords related to the global AIDS response including terms such as ‘National AIDS Fund’ and ‘Global Business Coalition’. The methodology and limitations of analyzing internal tobacco industry documents have been discussed elsewhere (MacKenzie, Collin & Lee 2003). Follow-up searches using a snowballing technique were conducted to further identify relevant industry actors (such as the Tobacco Growers’ Association of Malawi) and campaigns (such as the Virginia Slims Legends Tour) involved in AIDS-related activities. Then, the websites of the four largest tobacco companies’ (Philip Morris, BAT, Imperial Tobacco, and Japan Tobacco) were searched for the terms ‘HIV’ and ‘AIDS’. Reports on corporate social responsibility and charitable giving from these TTCs were correspondingly searched for any mention of HIV or AIDS.

The researchers then examined documents from AIDS organizations to identify policies related to engagement with TTCs. These queries were conducted first by searching the sites of those organizations mentioned in the tobacco industry documents (such as the Global Business Coalition and National AIDS Fund), and searching well-known global AIDS organizations’ (including UNAIDS, the Global Fund to Fight AIDS, TB and Malaria (GFATM), and Funders’ Concerned about AIDS) sites with the term ‘tobacco’ as well as the names of the largest tobacco corporations. In some cases, more specific online searches were conducted to verify reference to specific events or partnerships. Two authors reviewed all materials independently to ensure that relevant data were identified, interpreted appropriately and properly contextualized.

There were a number of limitations to the research. Many AIDS organizations have not kept historic policy documents or put them online. For example, records from some organizations, such as the Global Business Coalition on Health (GBC) and National AIDS Fund, from the 1990s (when both worked closely with the tobacco industry) were not available. The restriction to online searches meant that results were limited to what documents were made public. Furthermore, it is demonstrated that the TTCs destroyed many documents that should have been made available on the Legacy database (MacKenzie *et al*. 2003). While this restricts analysis of why policies changed, existing documents present rationales that, when contextualized within the broader AIDS and tobacco control histories, generated a comprehensive narrative.

## 3. Fair play? Philip Morris’ support for the early AIDS response in the USA

The initial response to AIDS in the USA during the 1980s was characterized by conflicts between affected communities – mostly gay men and their allies – and repressive public reactions. When infections first appeared among young homosexual men, homophobia combined with ignorance to generate a particularly oppressive and stigmatizing response (Fee & Krieger 1993). People Living with AIDS (PWA) were denied health care, lost their jobs, and were evicted from housing. Initial state action was slow and inadequate. US President Ronald Reagan did not mention the epidemic until 1985, and his first measures were to ban PWAs from entering the USA and to promote sexual abstinence (Fee & Krieger 1993).

AIDS advocacy and self-help groups sprang up across the country demanding more progressive policies and challenging homophobic and discriminatory attitudes. The Gay Men’s Health Crisis, founded in 1981 in New York City, was the first formal PWA support group, and throughout the 1980s, similar ASOs developed across the USA (Knight 2008). Membership largely consisted of middle-class white males, who were well educated, articulate, and professional. They rejected state-sponsored campaigns that characterized them as statistical risk groups and as populations of disease carriers (Fee & Krieger 1993). Instead, they argued that the epidemic was not the result of their sexual orientations or behaviors, but the homophobia, stigma, and discrimination that forces individuals to avoid information and medical care (Bayer & Fairchild 2006). They took to the streets, staging ‘die-ins’ in front of government and pharmaceutical offices and coordinating national protests.

**Fig. 1.**
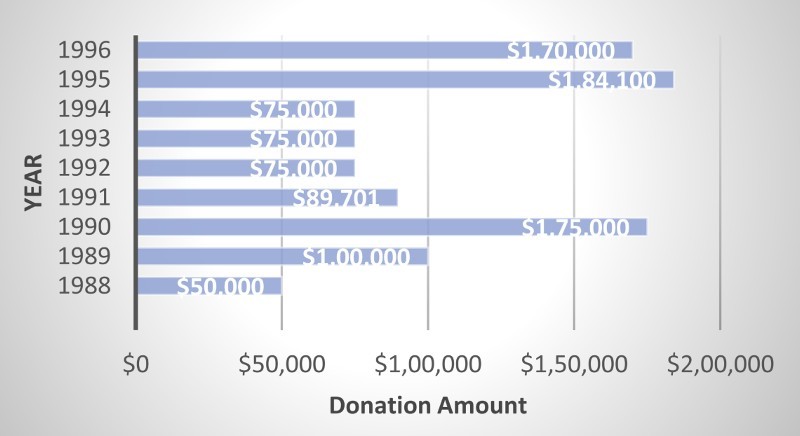
Philip Morris contributions to the National Aids Fund (Offe 1996).

In one such case, Act-Up, a political AIDS rights organization, decided to boycott Philip Morris Companies Inc. (PM), in 1990, when it found out that the TTC made donations to Senator Jessie Helms’ campaigns for re-election (McGovern 1987), as well as his personal charity, the Jessie Helms Center (Lewis 1989). Helms had promoted a particularly repressive response to AIDS, such as adding the Helms Amendment to the AIDS Appropriation Bill, which banned government funding for any AIDS education materials that ‘promote or encourage, directly or indirectly, homosexual activities’ (US Senate 1987, 1). Act-Up made three demands of PM: that it stop funding Senator Helms; that it renounce its past support for Helms; and that it meet with Act-Up representatives to discuss PM’s corporate responsibility to the LGBT community and AIDS response (Act-Up 1990; Offen 2005). In response, PM justified its donations to Helms as based on his representation of North Carolina, where PM ran a number of processing plants, as well as due to his support for the tobacco industry in general (PM 1992). Helms had opposed the increase of taxes on cigarettes, as well as other proposals to regulate the tobacco industry (Maguire 1993). PM refused to stop supporting Helms, but did increase funding to the AIDS response – illustrated by the temporary rise in donations to the National AIDS Fund in 1990 as depicted in Fig. 1. Having secured this additional funding for the AIDS response, Act-Up called off the boycott (Offen 2005; PM 1992).

Over the next decade, PM became one of the largest corporate donors to the National AIDS Fund. The fund had been created in 1988 to generate resources and provide support for ASOs and received donations from a variety of sponsors, including the Ford Foundation and Elton John Foundation (NAF 2007). One of the Fund’s signature projects was Positive Helpings, a nutrition program for PWAs. Between 1996 and 2001, Positive Helpings distributed more than US$ 3 million from PM to food and meals programs serving PWAs. In addition to supporting the National AIDS Fund, PM donated to a wide range of AIDS organizations, including the Gay Men’s Health Crisis, the Lutheran Church and the American Foundation for AIDS Research (PM 1995). PM framed its support to the AIDS response as an extension of the company’s contributions to the arts, stating:

For the Philip Morris family of companies, the AIDS epidemic of the 1980s hit close to home. Because of our support of the arts, we became aware of the devastating loss to the arts community wrought by the spread of this disease. (PM 2000, 2)

PM also explained support for AIDS nutrition programs, such as Positive Helpings, as an extension of Kraft’s (a food corporation that was at the time a subsidiary of PM) programs, which prioritized nutrition-related charitable giving.

However, PM also hoped to use its relationships with ASOs to foster competition for resources that were being directed toward tobacco control. As evidence mounted regarding the harms of smoking and second-hand smoke, the US government increased funding to civil society organizations to conduct anti-smoking youth education and tobacco control advocacy. PM used the networks it had established through its AIDS programs to contest this support. In 1996, the company initiated a strategy entitled ‘Fair Play’, which aimed to limit the effectiveness of tobacco control advocates. Fair Play documents reveal a proposal to mobilize social activists, including those fighting AIDS, to compete with tobacco control groups for government funds. As suggested by Joshua Slavitt, PM Issues Manager:

An additional option would be to identify other groups who feel they are not receiving adequate federal or state funding for their programs. Examples could include, public literacy programs, AIDS research, programs to help the physically challenged, etc … .This effort may lead to opportunities to encourage groups to divert federal or state funds away from tobacco control to other vital public programs during the appropriations process. (Slavit 1996, 9)

PM planned to achieve this through ‘work with Corporate Contributions and other internal departments to identify individuals or organizations that can act as “emissaries” to reach out to these groups’ (Slavit 1996). By letting AIDS and other organizations know how much state funding was going toward tobacco control groups, PM hoped to mobilize them to compete for funds and critique government priorities (PM 1999).

PM also needed allies to help it counter negative publicity and improve its corporate image. The Commissioner of the Food and Drug Administration testified before US Congress in 1994 that, despite TTC assertions to the contrary, nicotine was indeed a highly addictive drug in tobacco. Lawsuits against the industry, mounted by several US states and whistleblowers, led to the release of internal industry documents revealing how TTCs had attempted to obscure evidence about the harms and addictive elements of smoking. Scholarly analysis and media reporting based on the revelations from these documents severely damaged the reputation of the tobacco companies (Ashley 2003). By 2000, PM’s own research indicated that it was one of the least trusted corporations in the USA and that a reputation management strategy was urgently needed (PM 2000).

PM used AIDS fundraising events to improve its public image. For example, its largest AIDS fundraiser was the Virginia Slims Legends Tours – a series of tennis tournaments and all-female musical performances. The tour raised approximately US$ 4 million for the National AIDS Funds between 1995 and 1998 (Rosenberg & Siegel 2001). It also created opportunities for PM to share its message about smoking and the tobacco industry. PM provided instructions to representatives of the National AIDS Fund on how to respond to criticism about receiving tobacco funding. They were to state:

We’re proud of our relationship with Philip Morris U.S.A. and Virginia Slims, and we support their belief in freedom of choice. Philip Morris, through its diverse food, beer and tobacco companies, has been very supportive of organizations that are leading the fight against AIDS. They were among the first corporations to assist in AIDS research, AIDS prevention/education and direct care for people with AIDS. The Virginia Slims brand has established a history of its own, through various promotions, that have benefited AIDS research over the years. Let’s remember, AIDS does not discriminate and neither should we. (PM 1994, 1)

The statement used phrasing that sought to unite the AIDS response with smokers’ rights campaigns. The reference to freedom of choice refers to PM’s frequent argument that smokers had the right to ‘choose’ to smoke, with tobacco control policies threatening that right (Balbach, Smith & Malone 2005). In the context of an AIDS fundraiser, largely targeted at the LGBT community, this framing also reflected acceptance of sexual choice (Stevens, Carlson & Hinman 2004). Similarly, reference to discrimination refers to the stigma attached to the virus, and echoes earlier campaigns by PM that smoking regulations discriminate against people who smoke. For example, in a press release from PM, the Vice President of Corporate Affairs is quoted as arguing that businesses need to

at least think before taking action that will discriminate against a large segment of their employees [i.e. smokers], their customers, and their vendors … . The time has come when smokers will not sit passively and have their rights trampled. (PR Newswire 1988, 1)

PM’s language around AIDS subtly mirrored pro-tobacco messaging.

Such positive publicity also provided PM with lucrative marketing opportunities. Most American ASOs had strong connections to the LGBT community, who had, and continue to have, higher rates of smoking than the general public. A national household survey on drug abuse in 1990 found that 35% of gay men and 38% of lesbians had smoked cigarettes in the past month compared to 27% of men and 22% of women in the general sample (Drabble 2000). Studies conducted across a range of contexts suggest that LGBT populations have a higher smoking prevalence than the total population (Offen, Smith & Malone 2008). Higher smoking rates are attributed to the additional levels of reported stress and need for acceptance or desire for rebellion among LGBT youths. Smoking is also associated with the culture of ‘coming-out’ (Drabble 1999). As LGBT communities have been shown to prefer brands that support LGBT-related causes, supporting the AIDS response provided PM with a marketing advantage among a population group with high smoking rates (Drabble 2000; Goebel 1994).

Noting the specific characteristics of the LGBT market, PM not only targeted LGBT smokers in traditional marketing campaigns, but it also used AIDS initiatives to develop positive brand recognition (Smith & Malone 2003). Between 1998 and 2002, PM paid for advertisements in *Out Magazine* that listed the top ten ‘Companies That Care’ about LGBT community causes (Stevens *et al*. 2004). The 2002 advertisement put PM in the top spot and celebrated PM’s US$ 14 million in donations to AIDS-related organizations (Out Magazine 2002). In 2002, PM ran a similar advertisement in PRIDE.02, ‘the Magazine for Gay Pride in the USA’, which noted that PM was one of the largest corporate contributors to the fight against AIDS. Such publicity presented PM as an ally in the AIDS response, seeking to generate a favorable reputation, and, therefore, market share, within the LGBT community (Stevens *et al*. 2004).

During the 1990s, ASOs were initially grateful for PM’s support (Drabble 2000). In 1990, federal US funding for AIDS programs was just US$ 3.1 billion, which was grossly inadequate (Summers & Kates 2004). Most ASOs depended on donations from private individuals, churches, and charities to keep their doors open (Fee 1994). Tobacco industry funding was the largest corporate grant for many American ASOs. Furthermore, tobacco funding provided more flexibility than government and foundation grants, allowing ASOs to design programs as they saw fit, and tobacco grants had substantially fewer reporting requirements. Grants from PM could be used for capital and administrative expenses, which was not typical of other corporate or foundation funding (Stone & Siegel 2004). For example, PM provided money for general operating costs to the National Women and AIDS Project and Philadelphia Sciences Institute (PM 1995). In return, the AIDS response provided PM with opportunities to divert attention and resources away from tobacco control, and to enhance its market position and messaging.

## 4. Targeting WHO’s ‘blind spots’: TTCs use of AIDS as a diversion from global tobacco control

By the early 1990s, awareness was increasing of growing AIDS epidemics in low- and middle-income countries (LMICs). In particular, a generalized epidemic (as opposed to epidemics contained amongst specific population groups as was the case in most high-income countries) was emerging in sub-Saharan Africa. In South Africa, HIV prevalence among pregnant women climbed from less than 5% of the population in 1990 to nearly 30% in 1997 (UNAIDS 1998). As treatments for those infected, and to prevent HIV transmission from mothers to infants, was not yet available in LMICs, due to the high cost and lack of health resources, increasing HIV infections were followed by mortality, orphaning and further devastating social impacts (Poku 2006). AIDS epidemics in LMICs outside of sub-Saharan Africa were less severe but still caused widespread concern. AIDS rates in the Caribbean (1.82%) and South and South East Asia (0.61%) were lower than in sub-Saharan Africa (7.4%) but higher than in North America (0.55%) and Western Europe (0.23%). As a result, there were predictions of burgeoning generalized epidemics in LMICs and calls for a global response (Pisani 2008; UNAIDS 1998). Consequently, both the amount of funding for AIDS and number of organizations addressing the epidemic rapidly increased. Contributions from donor countries for the AIDS response rose from US$ 90 million in 1990 to US$ 1359 million in 2000 (Knight 2008). In 1996, the United Nations Joint Program on AIDS (UNAIDS) was formed, and by the turn of the millennium, discussions were underway to create innovative financing mechanisms for the AIDS response.

Much less attention was paid to the increasingly globalized use of tobacco products (Callard 2010). As countries in North America and Western Europe implemented tobacco control measures and education activities, throughout the 1990s, smoking rates in what had been the largest markets for TTCs began to decline. Consumption in the USA fell from approximately 750,000 cigarettes in 1981 to less than 500,000 in 2001 (WHO 2008). In response, TTCs’ shifted their focus to emerging markets in Asia, Africa, and Latin America, where there were weaker regulations, growing populations, and rising incomes (Lee 2014). Initially, TTCs faced few barriers in restructuring their operations toward this globally oriented strategy. Tobacco control was still seen as primarily a domestic concern. The WHO Tobacco and Health Unit, located under the Substance Abuse Program, collected and aggregated available data on tobacco use and its health consequences in members states, but was unable to do much else as it had only a handful of staff and a limited budget (Lee 2014). Meanwhile, domestic governments in LMICs were not overly concerned about increasing tobacco use. Though tobacco use was increasing in LMICs, the relatively delayed impacts of smoking (tobacco-related illnesses often do not appear until decades after habits begin) meant that tobacco use did not present an imminent health threat (WHO 2014). As many LMICs faced numerous health challenges related to lack of public health resources, and widespread poverty and infectious disease, tobacco control was not considered an urgent priority. As a result, TTCs were able to mitigate losses due to market contraction in high-income countries by increasing sales in LMICs.

To create awareness about the growing disease burden from tobacco use in LMICs, public health advocates began organizing international events and campaigns. The 8th World Conference on Tobacco or Health (WCTOH), held in Buenos Aires in 1992, was the first tobacco conference held in an LMIC. The conference was described by Muggli *et al*. as ‘a significant moment in globalizing tobacco control’, because it was the first meeting to concentrate on the dangers of second-hand smoke and the effects of tobacco use in LMICs (2008, 2). Conference organizers advocated the introduction of tobacco control measures in LMICs comparable to those adopted in high-income countries.

Fearful of how such policies might impact its market expansion in LMICs, BAT devised a planned to create a distraction from the WCTOH. Jorge R. Basso Dastugue, a representative from Nobleza-Piccardo, BAT’s subsidiary in Argentina, proposed a strategy to counter the WCTOH by capitalizing on the growing concern about AIDS:

Being the disease of the century and a preventive disease, AIDS should be ‘public enemy No. 1’ because of its terminal consequences at every age. Facing the [*sic*] AIDS increasing importance in the world and in Argentina we believe this disease to be the sole matter cable [capable] [*sic*] of eclipsing the [WCTOH] Conference. (Dastugue 1992, 3)

One of Dastugue’s strategy documents, entitled Proposed Possible Action To Take Against [the WCTOH], suggested, ‘have groups fighting against AIDS claim this conference for their priority as compared to tobacco’ (Dastugue 1992, 3). Documents described plans to generate competition for media and policy attention by sponsoring AIDS publicity events at the same time as the conference. Plans were proposed to bring in a ‘world known medical doctor’ to divert press attention, and to have publicity films on AIDS projected on large screens in different areas of the city at the same time as the WCTOH. To avoid questions about the timing of such campaigns, these promotions would not identify BAT as the sponsor. Instead, BAT funding would be channeled through a local AIDS foundation (Dastugue 1992).

While it appears that not all planned activities were carried out, BAT did sponsor music concerts and soccer matches promoting AIDS awareness in Argentina at the same time as the WCTOH (Muggli & Hurt 2003). The ‘world known doctor’, mentioned in Dastugue’s plan may have been Luc Montagnier, one of the discoverers of HIV, who visited Argentina at the same time as the WCTOH. The Buenos Aires newspaper, *La Penza* (1992), reported on Montanger’s visit, which coincided with the launch of the second phase of the National Program on AIDS by the Argentina Ministry of Health and Social Action. Such events fulfilled BAT’s goal of creating competition between AIDS and tobacco control for media and political attention.

Despite such efforts by TTCs to subvert tobacco control efforts, WHO continued to promote the idea of a global convention on tobacco control. At the World Health Assembly (WHA) in 1992, the concept of an international legal instrument for tobacco control was put forward (Roemer, Taylor & Lariviere 2005). In 1995, WHO member states adopted WHA Resolution 48.11 calling for ‘an international strategy for tobacco control’, including the adoption of this international instrument. In 1996, Resolution 49.17 was adopted at the WHA, calling on all parties to fast track negotiations of the FCTC. In 1998, WHO Director-General Gro Harlem Brundtland made global tobacco control one of two cabinet-level priorities and negotiations on the WHO FCTC commenced in 1999 (Lee 2014).

Alongside these developments, BAT maintained the message advanced previously in Argentina – that global health co-operation should prioritize AIDS, not tobacco control. In a document entitled, *The WHO Framework Convention on Tobacco Control: Our view*, BAT argued that the convention:

Would impose an additional burden on many governments, which have already determined their own domestic policy needs. There are likely to be serious resource implications for many national governments, particularly in developing countries, which have already committed stretched resources to health policy matters they consider higher priority, such as HIV. (BAT 2000, 2)

This messaging sought to discredit the WHO by questioning if its priorities aligned with those of LMICs. While AIDS was a growing concern in LMICs, as outlined above, in most countries outside of sub-Saharan Africa, there were arguably more pressing health matters as the majority of child and adult deaths continued to be caused by non-communicable diseases, many of which were related to the growing use of tobacco products, such as lower respiratory infections (WHO 2003). However, BAT capitalized on the growing panic around AIDS, to question WHO’s priorities and argue it neglected LMICs needs.

An important ally of this strategy was the industry front group the International Tobacco Growers Alliance (ITGA). Established in 1984 by the tobacco industry, the ITGA was a global agricultural lobby group on tobacco farming issues (Tobacco Tactics 2014). While presenting itself as an independent collective of tobacco farmers, the ITGA received substantial funding from TTCs to create resistance to tobacco control efforts (Rimmer 2005). In the late 1990s, BAT developed a plan to work with ITGA members in LMICs to lobby the WHO to direct greater attention to AIDS. A 1998 document outlines a strategy to, ‘Target WHO’s blind spots on key primary health priorities, such as AIDS prevention and malaria. Working through African and Latin American members, ITGA will build actions with their governments to put pressure on WHO’ (ITGA 1998, 4). To advance this strategy, ITGA members participated in AIDS-related events and championed the need for greater AIDS resources from the WHO. Shabanji Opukah, BAT’s Head of International Development Issues, wrote in an email that the ITGA Executive Director called him to report on the outcomes of a Southern African regional meeting:

The ITGA agreed to support fully a proposal for a pan-African aids conference to be held in Zambia in September hosted by the health ministry at which they will discuss the aids scourge in Africa. The ITGA is going to present what their grower associations have been doing to support government and NGO efforts in combating AIDS in Africa and through that highlight the importance of tobacco to the economy whilst relegating it as an issues [*sic*] in the health priorities of these countries. Then [*sic*] idea is to use the forum to challenge and ridicule the WHO convention. (Opukah 1999, 1)

By highlighting AIDS, the ITGA aimed to suggest that while the tobacco industry was addressing the epidemic and supporting the local economy, WHO priorities were misplaced, and the FCTC would not benefit LMICs.

To further propagate this message, in 1999, BAT paid for representatives from the ITGA to meet with representatives from the WHO regarding the FCTC (Opukah 1999, 1). An article about the meeting was published in the industry-sponsored *Tobacco Journal*. It quoted the ITGA President, Richard Tate, as noting that while the ITGA disagreed with WHO over tobacco control, it encouraged it to increase its commitments to addressing AIDS and Malaria: ‘Many of our members are from developing countries and know the suffering and distresses caused by both diseases. This is the type of fundamental public health campaign that we applaud’ (Hallmark 1999, 1). Another article on the meeting celebrated BAT’s contributions to the AIDS response in Southern Africa: ‘In Zimbabwe, for example, tobacco farmers have set up one of the most extensive AIDS prevention programs in rural areas. Without their action, there would have been few resources available to combat this terrible scourge’ (PR Newswire 1999, 1). This messaging showcased the contributions of the tobacco industry to the AIDS response, at the same time as suggesting WHO was not doing enough to address the epidemic because it was preoccupied with tobacco control.

The ITGA also championed AIDS at tobacco control-related events. In 2000, PM paid for ITGA representatives from Malawi to attend a UN Economic and Social Council meeting on the FCTC. The Malawian delegates listed their goal in attending the meeting as to get the WHO to ‘reassess importance of tobacco growing for developing countries; put health priorities on real world basis, and go public where good work is taking place’ (ITGA Malawi 2000, 2). The concept of refining health priorities ‘on a real world basis’ reiterated the message that tobacco control was a concern primarily in high-income countries, while LMICs suffered from other health concerns, such as AIDS, which should be prioritized by WHO because of these countries’ relative reliance on external assistance to address public health needs. The delegates reported to PM that they had advised the Malawian government representatives to present a similar message. Indeed the government of Malawi’s subsequent statement on the FCTC, which was included in communications between the ITGA and PM as evidence of influence, expressed the belief ‘that the deaths due to Tobacco, for example, in Malawi are exaggerated, ignoring more important problems of Malaria and H.I.V. Aids’ (quoted in ITGA Malawi 2000, 4). PM and BAT used networks with AIDS organizations and the ITGA to foster a discourse that capitalized on growing concerns about the AIDS epidemic at the expense of tobacco control.

## 5. ‘A pact with the devil’: restricting TTC’s corporate social responsibility opportunities

As evidence emerged on the higher smoking rates among LGBT populations in the USA (as outlined above), AIDS organizations began questioning the motivations behind tobacco industry funding. The Coalition of Lavender Americans on Smoking and Health was supported by the Center for Disease Control, in 1996, to organize a conference, ‘Alive With Pleasure!’, which focused on the prevention of tobacco use and alcohol problems in the LGBT communities. The Coalition met with LGBT and AIDS organizations to discuss the health impacts of smoking and suspect practices of TTCs (Drabble 1999). Their concerns were augmented by research showing that smoking was particularly harmful to PWAs and could impede treatment effectiveness (Furber, Maheswaran, Newell & Carroll 2006). The findings created a link between anti-smoking campaigns and AIDS health care. Rather than constructing a choice between tobacco control or AIDS, both issues were recognized as relevant health issues for ASOs and LGBT groups.

In response to growing awareness of smoking-related diseases within the AIDS community, as well as wider public attention to the public relations tactics of tobacco companies, many ASOs, and LGBT organizations demanded that their boards refuse tobacco-related funding. As early as 1991, a leader of Act-Up San Francisco argued in an editorial, published in the Gay Community News, that receiving funding from PM, ‘represents us taking money from walking over the bodies of those killed by cigarettes … I would characterize this as a pact with the devil’ (as cited in Offen, Smith & Malone 2003, 205). By the late 1990s, such opinions had become widespread with the Coalition of Lavender Americans on Smoking and Health arguing, ‘We gay adults have a responsibility to start treating tobacco as a serious threat to our community. It’s not some minor concern to be ignored while we focus on AIDS and breast cancer’ (Drabble 1999, 8). In 1998 and 1999, the Coalition produced ‘The Ethics of Tobacco, Alcohol, & Pharmaceutical Funding: A Practical Guide for LGBT Organizations’, which advised against accepting tobacco funding, arguing that it was a harmful form of indirect marketing. The Coalition encouraged ASOs to develop guidelines on what type of corporate funding they welcomed, and under what conditions. The Gay and Lesbian Medical Association developed, in 1998, one of the first such policies, stating that: ‘The Gay and Lesbian Medical Association will not accept direct funding from alcohol or tobacco manufacturers or distributors’ (quoted in Drabble 2000, 3). While not all AIDS-related organizations made the choice to refuse TTC funding, it became increasingly common. A survey of ASOs in the USA found that the most common reason for refusing TTC funding was recognition that accepting tobacco money conflicted with organizational mandates related to improving population health, and due to concerns about public association between the organization and tobacco corporations – due to TTC’s increasingly poor reputation (Stone & Siegel 2004). By linking health issues, ASOs recognized that tobacco control initiatives were not in competition with AIDS programs, as TTCs had suggested but had similar overall health goals, which TTCs threatened.

It is notable that many ASOs adopted policies against receiving tobacco funding relatively early, compared to other charitable sectors; in developing such early policies, ASOs were earlier identifiers of the ethical issues raised by partnerships with TTCs. ASOs were able to demonstrate leadership regarding the ethics of accepting TTC funding for three reasons. First, as noted above, ASOs began to recognize that the holistic well-being of PWAs required consideration of health issues beyond those specifically related to the virus. Secondly, AIDS organizations benefited from an extensive global network of both grassroots organizations and global institutions that helped to disseminate concerns about the tobacco industry and resulting policies. Finally, by the late 1990s, resources for AIDS were increasing, rising from US$ 292 million spent globally in 1996 to US$ 4630 million in 2003 (Knight 2008). Therefore, ASOs had access to more resources and greater flexibility around with whom they could partner.

Rejection of tobacco funding trickled up from ASOs and partner organizations to global forums. During a 2002 retreat to establish the private sector delegation to the GFATM Board, it was agreed that TTCs should not be allowed to be members. Meeting minutes from the retreat note ‘there are certain industry sectors that could create conflict, such as tobacco industry representatives’ (GFATM 2002, 2). In 2009, the GFATM formalized and expanded this policy, passing a board resolution that prohibited the fund from receiving donations or working in partnerships with TTCs (GFATM 2010). UNAIDS developed ‘Guidelines on Working in Partnership with the Private Sector’, which recommend ‘not developing partnerships with entities in sensitive sectors such as tobacco, gambling, and pornography’ (2007, 1). This stance was reiterated in a 2013 UNAIDS Lancet Commission Discussion paper on ‘How Should the Global Health and AIDS Architecture Be Modernized for the Post-2015 Development Agenda?’ which noted the growing role of TTCs in LMICs, and the need to protect against this influence. It reads, ‘Public health goals, however, need to be safeguarded against private sector strategies, such as the efforts of tobacco companies to enlarge their markets in low- and middle-income countries’ (2013, 7). AIDS organizations were some of the first to advance policies against TTC interference in global health forums.

In 2011, theGBC decided to prohibit membership to parties with connections to the tobacco industry. It released a statement that read, ‘The membership of tobacco companies and their parent companies in the Coalition would be inconsistent with, and would contradict GBC’s mission and vision.’ This was significant as both PM and BAT had been longtime members of the Coalition. The Coalition developed this policy the same year it changed its name from the ‘Global Business Coalition on AIDS, TB, and Malaria’ to ‘Global Business Coalition on Health’. As the organization widened its focus to broader health goals, the divisions between health issues such as AIDS and tobacco-related illnesses blurred. It became intolerable for a health coalition to include members who profited from products that contributed to one of the most pressing global health crisis.

The GBC also noted that it had made the decision to restrict tobacco industry membership in order to align its policies with those of the WHO’s FCTC, which strictly limited interactions and partnerships with the tobacco industry. The FCTC, signed by 168 states in 2003, included Article 5.3 on ‘the protection of public health policies with respect to tobacco control from commercial and other vested interests of the tobacco industry’, which recommended parties, ‘denormalize and, to the extent possible, regulate activities described as “socially responsible” by the tobacco industry, including but not limited to activities described as “corporate social responsibility”’. Although the FCTC, as an intergovernmental treaty, was only binding for signatory states, measures regarding industry influence were adopted by non-state actors, including AIDS organizations, as justification for terminating partnerships with TTCs. The legitimacy of the convention, in turn, was strengthened by compliance of these organizations. Instead of AIDS distracting from the FCTC, as TTCs had hoped, the FCTC increased awareness among AIDS organizations about the need to limit TTC engagement and provided a framework through which to do so.

Concurrently, a number of prominent funders developed supportive policies to limit TTC involvement in global health. The Bill and Melinda Gates Foundation, one of the largest private donors in the global health field, established a policy of not providing funding to organizations that accepted resources from or had connections with the tobacco industry (BMGF 2010). As the Gates Foundation also funded tobacco control initiatives, it noted that accepting funding from or working in partnership with organizations with links to the tobacco industry would be hypocritical to broader global health goals. In 2010, the foundation pulled funding from the International Research and Development Center (IDRC) – a research institute in Canada that supports organizations in LMICs to implement both AIDS and tobacco control programs – when it found out the Executive Director was also on the Board of Imperial Tobacco Canada. The Gates statement read:

The foundation was recently informed that the chair of the board of our partners, the International Development Research Centre (IDRC), has until recently also been a Director of Imperial Tobacco Canada, Ltd. We are deeply disappointed by this revelation and feel this conflict is unacceptable as we work to support meaningful tobacco control programs in Africa. (BMGF 2010, 1)

This case, which was widely covered in the media, propagated the message that in order to access Gates resources, organizations needed to sever all ties with the tobacco industry (see, e.g., Blackwell 2010; Market Wire 2010). As the largest private donor to the global AIDS response, donating US$ 2.5 billion to AIDS programs in 2014, the Gates Foundation dwarfed TTC funding and could set the terms of partnerships for AIDS-related organizations.

The widespread rejection of tobacco money by civil society organizations, institutions, and other private actors in the AIDS response disseminated through global networks. Imperial Tobacco reported dismay, in 2005, when a West African charity it had historically provided funding to for AIDS programs refused its money. The TTC wrote in its Social Responsibility Report:

Our efforts for further and more widespread action on AIDS have been impeded by the difficulty of finding a suitable partner to help us. We were saddened that the head office of an international NGO prevented their regional office from partnering us in several developing countries, solely on the grounds of accepting money from a tobacco company. (Imperial Tobacco 2005, 1)

Overall, by the late 1990s onwards, AIDS organization in the USA and then globally turned away from tobacco funding. This was initially prompted by evidence of the harmful health effects of tobacco use on PWAs but eventually was motivated by recognition of TTCs’ use of the AIDS response to further its interest, and supported by the development of shared norms and greater resources. AIDS organizations were early recognizers of the ethical problems of partnerships with TTCs. Their stance in turn proved supportive of the FCTC process and adoption of measures limiting industry influence in health programs and policy.

## 6. TTCs and AIDS in sub-Saharan Africa

However, TTCs have not been completely shut out of the AIDS response. By 2007, Imperial Tobacco had found another AIDS organization – a French charity called Cinomade – to support in Cote d’Ivore, and continues to fund AIDS programs in sub-Saharan Africa (Imperial Tobacco 2007). BAT sponsors AIDS programs in Kenya, Nigeria, and South Africa (ASH 2007; BAT 2014). Japan Tobacco funds AIDS programs in South Africa and Tanzania (Japan Tobacco 2013), and Philip Morris gives extensively to AIDS projects in South Africa (PM 2010, 2011).

TTCs have continued to be active supporters of the AIDS response in sub-Saharan Africa, despite resistance from some AIDS organizations and global policies discouraging such partnerships, for two reasons. First, the needs of AIDS initiatives in sub-Saharan Africa remain greater than available resources. Global funding for the AIDS response is projected to be $21.7 billion by the end of 2015, still short of the $22 to $24 billion required to fund programs in LMICs (UNAIDS 2014). As a result of stagnated global health resources and continued insufficient domestic resources, many AIDS organizations remain desperate for funding (UNAIDS 2014). Secondly, many sub-Saharan African governments have not adopted comprehensive policies to restrict TTC influence. Some states, like Malawi and Zambia, have not signed and ratified the FCTC. Others, such as South Africa and Kenya, while imposing regulations on tobacco product advertising and marketing, do not regulate tobacco industry corporate social responsibility activities (ASH 2007). The combination of dire need and relatively weak regulations in many sub-Saharan African countries allows TTCs to continue to capitalize on the AIDS response.

TTCs continue to use AIDS projects to improve their image and reputation in sub-Saharan Africa and have even been celebrated for their contribution to the African AIDS response. In 2007, BAT sponsored an African–Middle East Conference on AIDS. At the conference, the Nigerian Minister of Labor praised BAT’s national workforce policy on AIDS and called on other businesses to implement similar policies (John 2007). Similarly, the Tanzanian Cigarette Company (a subsidiary of Japan Tobacco International) received a ‘Best Employer’ award in 2010, for AIDS and related programs for workers’ health. With no recognition of the contradiction of giving a tobacco company a health-related award, Japan Tobacco was commended by the Minister of Defense and National Security, and the Minister of Labor, Youth and Employment (Bitekeye 2010). It can be argued that such events are contrary to Article 5.3 of the FCTC, which both Nigeria and Tanzania have ratified because they create venues for government officials and TTC representatives to interact, failing to ‘de-normalize’ relationships between the government and tobacco industry (a recommendation under Article 5.3). TTCs use such events and awards to enhance their reputation as corporate citizens and to indirectly promote tobacco products despite advertising bans.

The continued participation of TTCs in the sub-Saharan African AIDS response demonstrates that efforts to shut tobacco money and influence out have not been comprehensive. TTCs have redirected their resources where the AIDS response remains desperate for funding and where government tobacco control policies are weak. While TTCs claim to be supporting the AIDS response in sub-Saharan Africa because of the dire need, and out of the desire to ensure the health of their employees, their past practices of using the AIDS response to subvert tobacco control efforts and market their products raise questions about the motivations behind their current AIDS programs.

Such questions are especially pertinent as rates of tobacco use are on the rise in many sub-Saharan African countries. Cigarette consumption on the continent almost doubled from just over 40 billion in 1990 to nearly 80 billion in 2010 (Blecher & Ross 2011). A greater portion (9%) of boys smoke than in other developing regions, such as South East Asia, and a greater portion of youth (both boys and girls) smoke compared to adults in sub-Saharan Africa, indicating that smoking prevalence is likely to increase over the next decade (Blecher & Ross 2011). Such trends suggest that tobacco control efforts remain essential to preventing greater tobacco-related illness and death in a region already struggling to address numerous other health challenges.

## 7. Conclusion

During the early AIDS response in the USA, PM provided funding to ASOs to support marketing campaigns targeted toward LGBT communities, to enhance the company’s reputation during a time of declining public trust of TTCs, and to support arguments for the diversion of resources from tobacco control initiatives. TTCs then applied a similar strategy at the global level as the FCTC process progressed. TTCs capitalized on growing concerns about the AIDS epidemic in LMICs – markets they were also expanding into – to argue that WHO should focus on AIDS, not tobacco control. TTCs aimed to generate competition between the two health issues, for resources and policy attention, to protect their interests from stronger regulation.

However, the collective decision by many AIDS organizations to refuse TTC funding, and thus the use of the AIDS epidemic to the tobacco industry’s advantage, demonstrates the capacity of health networks to restrict negative or co-opting influences through closer co-operation. ASOs used knowledge, of the harms of smoking for PWAs and the suspect practices of TTCs, to justify the termination of partnerships with TTCs. Through global networks and the creation of common policies, AIDS organizations thus pioneered practices that restricted the ability of TTCs to exert undue influence over global health policy. These actions by ASOs were supported by the availability of alternative funding sources and an agreed global governance instrument. The FCTC, while not binding on non-state actors, provided a set of shared norms which informed the actions of the AIDS community. Where the gap between resources for AIDS and health needs remains wide, and tobacco control policies are still relatively weak, however, TTCs remain actively involved in the AIDS response. In sub-Saharan Africa, notably, government ministers and TTC representatives still share the same podiums to congratulate each other on their AIDS efforts.

Evidence of tobacco industry involvement in the AIDS response raises a number of policy implications for both the AIDS and tobacco control communities, as well as broader concerns regarding the strengthening of global health governance. First, it should be recognized that competition among health issues can be sown and exploited by vested interests for reasons that run contrary to public health goals. TTCs used the AIDS epidemic to try to divert resources and attention away from tobacco control. Priority setting and the allocation of scarce resources should be undertaken according to evidence-based criteria and transparent processes. Moreover, there is a need for more collaborative, rather than competitive, approaches to increase societal resources for health needs overall.

Second, other public interest organizations can learn from, and build upon, the policies prohibiting funding from TTCs pioneered by AIDS organizations. TTCs continue to offer donations to a diverse range of charitable organizations concerned, for example, with the environment, domestic violence, poverty alleviation, arts, sports, and education (BAT 2014; PM 2010). These donations provide TTCs with a degree of legitimacy and reputational rehabilitation, while providing nontransparent means for interacting with policy-makers. The recipients of such funding need to be aware of the impact of TTC activities on public interests, industry tactics in seeking to influence public policy, and the consistency of these efforts with their own mandates and normative frameworks. Public interest organizations might look to AIDS organizations for best practices regarding ethical partnerships. Further research is needed on the extension of these best practices to minimize inconsistencies in practice across public health issue areas.

Third, solidarity across health issues requires mutually supportive approaches embedded in global governance arrangements. In this case, AIDS organizations adopted Article 5.3 of the FCTC which calls for the exclusion of tobacco industry participation in policy-making. AIDS organizations were able to review their own practices around TTC funding because they had a shared normative framework to draw from. Conversely, tobacco control efforts should recognize the particular needs of PWAs. As AIDS organizations have broadened their mandates, such as the Global Business Coalition on HIV/AIDS becoming the Global Business Coalition for Health, there is an even greater need for policy coherence.

Finally, to better enable AIDS organizations to choose not to accept TTC funding requires access to alternative resources. TTC funding for the AIDS response, notably in resource-scarce settings such as sub-Saharan Africa, requires replacement by other donors. Their funding could be provided with the condition of compliance with Article 5.3 to ensure that the needs of those affected by HIV/AIDS are not met at the expense of furthering the health-harming activities of TTCs. Such coordination would benefit the AIDS response, tobacco control initiatives and, most importantly, those individuals and communities worldwide affected by either major cause of ill-health and death.
